# Molecular insights into antimicrobial resistance in human bacterial pathogens: mechanisms, resistance genes, and translational diagnostic applications

**DOI:** 10.3389/fmicb.2026.1842688

**Published:** 2026-06-26

**Authors:** Nazmul Hossen, Maria Teresa Mascellino

**Affiliations:** 1 Department of Para Clinical Science (Microbiology and Public Health), Faculty of Veterinary Medicine and Animal Science, Habiganj Agricultural University, Habiganj, Bangladesh; 2 Department of Public Health and Infectious Diseases, Sapienza University of Rome, Rome, Italy

**Keywords:** AMR surveillance, antimicrobial resistance, mobile genetic elements, molecular diagnostics, whole genome sequencing

## Abstract

Antimicrobial resistance (AMR) represents one of the most critical global public health challenges. This review provides a comprehensive overview of the molecular foundation of AMR in human bacterial pathogens, including the biology of resistance genes and the importance of the mobile genetic elements—plasmids, transposons, and integrons—in facilitating the rapid horizontal transfer of resistance determinates across the populations. We critically evaluate current and emerging molecular diagnostic platforms — including targeted polymerase chain reaction (PCR), whole-genome sequencing (WGS), clustered regularly interspaced short palindromic repeats (CRISPR)-based technologies, and metagenomics — emphasizing their comparative performance, limitations, and suitability for point-of-care deployment. The review addresses the translational integration of molecular diagnostics into antimicrobial stewardship programmes and real-time AMR surveillance, with particular attention to the persistent gap between laboratory-generated genomic data and actionable clinical decision-making. Emerging evidence suggests that artificial intelligence (AI) and machine learning hold considerable promise for improving resistance phenotype prediction from genomic data and informing personalized antibiotic therapy, although widespread clinical implementation remains in its early stages. The transition from phenotypic to genotypic strategies represents a significant paradigm shift in AMR, with the potential to substantially improve surveillance, diagnostic accuracy, and therapeutic outcomes, provided that outstanding barriers in infrastructure, standardization, and equity are addressed.

## 1 Introduction

The invention of antibiotics in the early 20th century changed modern medicine by making deadly bacterial infections treatable. However, the emergence and worldwide spread of antimicrobial resistance (AMR) now places this major component of medical development in jeopardy (Aminov, 2010). The global research on antimicrobial resistance estimated that 4.71 million deaths were associated with bacterial antimicrobial resistance (AMR) in 2021, of which 1.14 million were directly attributable to resistant infections (GBD 2021 Antimicrobial Resistance Collaborators, 2024).

The enormity of this loss of life emphasizes the importance of developing novel technologies for the detection, monitoring, and management of AMR (Ahmed and Alghamdi, 2026; Chindelevitch et al., 2022). Clinical microbiology has depended on traditional methods for testing the susceptibility of bacteria to antimicrobial susceptibility test (AST), the determination of whether a bacterial isolate is inhibited by a given antimicrobial agent at clinically relevant concentrations. Standardized methods, such as disc diffusion, broth dilution, and gradient diffusion tests, have formed the basis of the clinical gold standard, providing valuable phenotypic data on the ability of bacterial growth to inhibit the agent being tested (Gajic et al., 2022; Salam et al., 2023).

**FIGURE 1:**
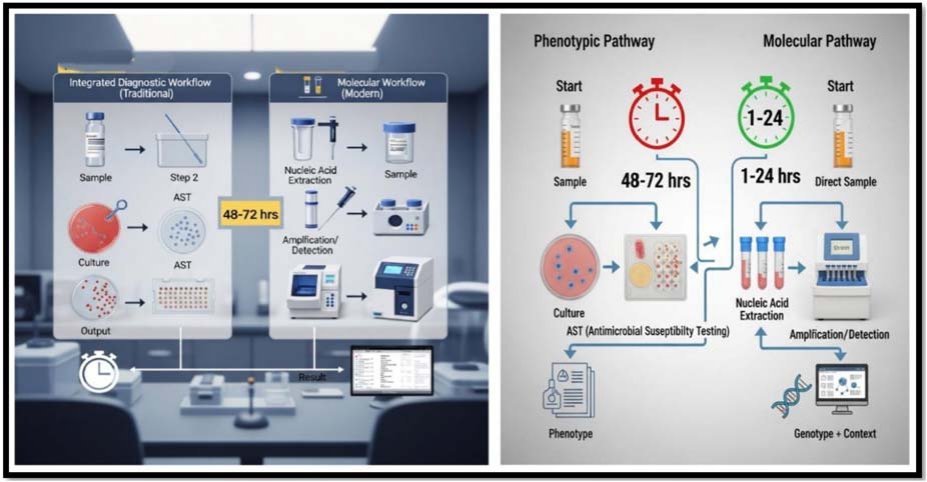
Integrated diagnostic workflow from sample to result. The following Figure provides a side-by-side comparison of the two processes of culture and AST to genetic detection. It shows that there is a significant difference in time between the two methods and shows the overall goal of both types of tests. A flowchart comparing the parallel phenotypic (culture → AST, 48–72 h) and molecular (direct sample → nucleic acid extraction → amplification/detection → result, 1–24 h) pathways, highlighting the significant time savings and different information outputs (phenotype vs. genotype + context). Created in Quill bot (2026) https://quillbot.com.

While the disk diffusion method is the gold standard AST, it usually requires 24–72 h to produce a definitive result. Furthermore, traditional AST provides limited information about the specific molecular mechanisms underlying resistance or the potential for inter-strain spread (Alviset et al., 2023). The emergence of molecular biology techniques has fundamentally transformed the ability to detect, characterize, and respond to AMR at the genomic level, opening new diagnostic paradigms with the potential to overcome these limitations. Figure 1 depicts the diagnosis workflow starting from sampling to the result.

The first cloning and characterization of the β-lactamase gene *blaTEM-1* is widely regarded as marking the beginning of the molecular era of antimicrobial resistance (AMR) research (Singh et al., 2018). Over the next several decades, the rate of resistance gene discovery has increased. For instance, polymerase chain reaction (PCR) technology began to be used as a method to amplify and identify resistance genes, enabling rapid, targeted detection of specific antibiotic resistance determinants in clinical and environmental isolates (Alsuof et al., 2025). This was followed by the introduction of next-generation sequencing (NGS), which allows the discovery of novel resistance-gene variants and their genetic contexts (Li et al., 2025). Currently, with resources such as the Comprehensive Antimicrobial Resistance Database (CARD) and ResFinder, researchers have access to curated collections of thousands of resistance genes, their variants, and the diverse genetic environments in which each can occur, greatly facilitating genome- and metagenome-based AMR surveillance (Li et al., 2025).

Despite these technological advances, the majority of clinical laboratories worldwide—particularly in low- and middle-income countries (LMICs)—continue to rely on phenotypic antimicrobial susceptibility testing due to constraints in cost, infrastructure, availability of trained personnel, and assay standardization (Iskandar et al., 2021). Understanding the functional relationship between specific antimicrobial resistance (AMR) genes and their corresponding antibiotic targets is not merely an academic concern. It is directly relevant to the selection of empirical therapy, decisions regarding de-escalation, and the accurate interpretation of molecular diagnostic results (Nusrat et al., 2025; van Hoek et al., 2011).

Resistance gene characterization therefore underpins all clinical applications of molecular diagnostics discussed in this review. This review critically evaluates the scientific trajectory from initial resistance gene characterization to the development of clinically deployable molecular diagnostic tests, highlighting key advances, persistent challenges, and emerging opportunities for improved AMR management.

The literature was retrieved from PubMed, Google Scholar, and Scopus using combinations of the following terms: “antimicrobial resistance,” “mobile genetic elements,” “resistance genes,” “molecular diagnostics,” “whole-genome sequencing,” “CRISPR diagnostics,” “antibiotic stewardship,” and their MeSH equivalents. Priority was given to peer-reviewed original research articles, systematic reviews, and meta-analyses published in English with a clear mechanistic or clinical focus. Studies lacking a defined mechanistic rationale, published in non-peer-reviewed venues, or identified as predatory publications were excluded.

## 2 The resistance gene arsenal: classification and mobiloma

### 2.1 Intrinsic vs. acquired resistance mechanisms

Bacterial resistance to antimicrobials operates through two fundamental mechanisms: intrinsic (or innate) resistance and acquired resistance. Intrinsic resistance represents a constitutive, species- or genus-specific property that is stably encoded in the bacterial chromosome and confers a baseline reduction in susceptibility to particular antimicrobial classes without prior exposure to those agents (Cox and Wright, 2013; Martinez, 2014). This form of resistance is predictable, phylogenetically defined, and reflects natural physiological or structural traits rather than recent genetic adaptation.

Intrinsic antibiotic resistance in Gram-negative bacteria arises largely from the outer membrane (OM), which acts as a selective permeability barrier due to lipopolysaccharide-rich lipid A and tightly packed hydrocarbon chains that reduce fluidity and slow drug influx (Obst et al., 1997; Vance and Vance, 1996). Narrow porin channels further restrict antibiotic entry based on size, hydrophobicity, and charge, contributing to intrinsic insusceptibility to many agents (Benz and Bauer, 1988; Cowan et al., 1992; Nikaido, 2003). In *Pseudomonas aeruginosa*, the OM’s low permeability—combined with periplasmic β-lactamase activity—confers marked intrinsic resistance to β-lactams (Hancock and Brinkman, 2002; Nakae et al., 1999). Efflux pumps, especially RND-type tripartite systems such as MexAB-OprM in *P. aeruginosa* and AcrAB-TolC in *Escherichia coli*, actively export diverse antibiotics using the proton motive force (Marquez, 2005; Poole, 2004). In Gram-positive bacteria, the thick peptidoglycan layer imposes a high permeability threshold for small molecules, rendering them intrinsically more susceptible than many Gram-negative species (Randall et al., 2013; Scherrer and Gerhardt, 1971). Overall, intrinsic resistance reflects the interplay of outer-membrane impermeability, efflux activity, and, in some cases, periplasmic inactivating enzymes such as β-lactamases, rather than a single isolated mechanism.

In contrast, acquired resistance arises when a bacterial strain develops resistance through genetic alteration, typically via chromosomal mutation or, more frequently, through acquisition of exogenous genetic material via horizontal gene transfer (HGT). Acquired resistance is dynamic, often inducible, and can spread rapidly across bacterial strains and ecological niches, contributing significantly to the emergence of multidrug-resistant (MDR) pathogens in clinical settings (Holmes et al., 2016).

Key examples of acquired resistance determinants include *bla*NDM-1 and *bla*KPC, conferring high-level carbapenem resistance in *Klebsiella pneumoniae* and other Enterobacterales; *mecA* and its variants such as *mecC* (Idrees et al., 2023), which encodes an altered penicillin-binding protein (PBP2a) mediating methicillin-resistant *Staphylococcus aureus* (MRSA) and broad resistance to most β-lactams; *vanA/vanB* gene clusters that confer acquired glycopeptide resistance in *Enterococcus faecium* through modification of the peptidoglycan precursor target; and *qnrS* and related genes, which encode plasmid-borne quinolone-resistance-determining region-protecting proteins that reduce fluoroquinolone susceptibility in *Escherichia coli* and other Enterobacteriaceae (Boralli et al., 2023; Liu et al., 2024; Rezazadeh et al., 2016; Woodford et al., 1997). The acquisition of mobile genetic elements (MGEs)—such as plasmids, transposons, and integrons—carrying pre-assembled resistance cassettes is the primary driver of rapid dissemination of acquired MDR phenotypes across healthcare facilities and geographic regions (Nodari et al., 2023).

### 2.2 Mobile genetic elements: the vehicles of AMR spread

The mobilome, a group of MGEs, plays an essential role in the AMR problem. MGEs allow for the ability to exchange resistance genes between different bacterial species at various taxonomic levels, including genus and strain levels.

Conjugative plasmids are extrachromosomal, self-replicating DNA elements that represent the most epidemiologically significant vehicles for resistance gene dissemination across species barriers. The IncF plasmid family, for example, carries *bla*CTX-M-15—encoding an extended-spectrum β-lactamase (ESBL)—predominantly in *E. coli* ST131, contributing to its global pandemic success (Agyekum et al., 2016; Carattoli, 2009). Inc_*X3*_ plasmids carrying *bla*NDM-1 have been identified in *K. pneumoniae* and *Enterobacter* spp. across multiple continents. The plasmid-mediated colistin resistance gene *mcr-1* was first identified on conjugative plasmids in *E. coli* of animal origin and has since disseminated globally (Bilal et al., 2021; Elshamy et al., 2023).

Furthermore, transposons, which are often termed “jumping genes,” are mobile DNA elements flanked by insertion sequences (IS) that enable movement within and between replicons. Composite transposons carry resistance genes flanked by IS elements on both ends, while the Tn3 family employs a resolution-based mechanism (Nicolas et al., 2015; Shkumatov et al., 2022). Tn1546, a Tn3-family transposon, has been instrumental in the global dissemination of the *vanA* gene cluster in vancomycin-resistant *Enterococcus faecium* (VRE). Transposons such as Tn21 carry integrons that further expand their resistance gene capacity in *Pseudomonas aeruginosa* and Gram-negative pathogens (Oskoui and Farrokh, 2010; Yu et al., 2003).

Integrons are genetic platforms that capture and express mobile gene cassettes via site-specific recombination mediated by the integrase gene *intI*. Class 1 integrons are the most clinically prevalent and are associated with arrays of resistance cassettes encoding resistance to aminoglycosides (*aadA*), trimethoprim (*dfr*), and β-lactams (*bla*; Deng et al., 2015; Fluit and Schmitz, 1999). Class 1 integrons are commonly found in *P. aeruginosa*, *Acinetobacter baumannii*, and members of the family Enterobacteriaceae, where they contribute to the simultaneous acquisition of resistance to multiple antibiotic classes (Lacotte et al., 2017).

The integrative conjugative elements (ICEs) are large mobile genetic elements (MGEs) that integrate into the host chromosome but can also excise and transfer to a new host via conjugation. They typically carry combinations of virulence factors and resistance genes (Rodríguez-Blanco et al., 2012). The staphylococcal chromosomal cassette *mec* (SCC*mec*) in *Staphylococcus aureus* carries the *mecA* gene encoding the low-affinity penicillin-binding protein PBP2a, which underlies methicillin resistance in MRSA; different SCC*mec* types (I–XII) have distinct epidemiological associations—SCC*mec* type IV is associated with community-acquired MRSA (USA300), while types II and III are more prevalent in healthcare-associated strains (Chambers and Deleo, 2009; Yamaguchi et al., 2020). Table 1 summarizes the major mobile genetic elements and their role in AMR dissemination.

**TABLE 1:** Major mobile genetic elements in AMR transmission.

Elements type	Key features	Example resistance gene	Transfer mechanism	Role in AMR dissemination	Reference
Plasmids	Circular extrachromosomal dsDNA; often self-replicating and variable in size, carrying resistance islands and other MGEs	*blaCTX-M, blaNDM, mcr-1, qnr* are all commonly plasmid-associated, but not exclusive to plasmids	Conjugation or mobilization	Major vehicles for epidemic and interspecies spread of AMR genes; important across clinical and ecological settings	Fursova et al., 2022; York, 2024
Transposons	Mobile DNA segments that can move between chromosome and plasmids; often associated with insertion sequences and composite structures	*vanA, blaVIM, aac(6′)-Ib* are valid examples of transposon-associated resistance determinants	Transposition within or between replicons	Mobilize resistance genes between chromosomes and plasmids and help build complex resistance regions	Babakhani and Oloomi, 2018; Noel et al., 2022; Shintani et al., 2023
Integrons	Gene capture and expression systems that collect gene cassettes by site-specific recombination	*aadA*, *dfr*, and some *bla* cassettes are appropriate examples	Site-specific recombination mediated by integrase	Promote assembly of multidrug resistance arrays by accumulating resistance cassettes	Akrami et al., 2019; Bhat et al., 2023; Partridge et al., 2009
ICEs	Chromosomally integrated elements that excise and tran5sfer by conjugation; also called integrative and conjugative elements	*Tet(M), erm(B), mecA* are reasonable examples of ICE-associated resistance genes in many bacteria	Excision followed by conjugative transfer	Enable stable chromosomal maintenance and spread of resistance and sometimes virulence traits	McLellan et al., 2022; Zheng et al., 2023

### 2.3 High-risk clones and global dissemination

Certain bacterial lineages—termed “high-risk clones”—have achieved global dominance through the convergent acquisition of antimicrobial resistance (AMR), enhanced virulence, and superior transmission fitness. The epidemiological impact of these clones is substantial, with several lineages responsible for the majority of multidrug-resistant (MDR) infections in healthcare settings worldwide (Mathers et al., 2015; Woodford et al., 2011).

*Escherichia coli* Sequence Type (ST) 131 is one of the leading extraintestinal pathogenic *E. coli* (ExPEC) lineages globally, particularly its H30 subclone, which carries blaCTX-M-15 on stable IncF plasmids. ST131 accounts for the majority of fluoroquinolone-resistant and ESBL-producing *E. coli* urinary tract and bloodstream infections worldwide (Johnson et al., 2016; Matsumura et al., 2016). A systematic study estimated that ST131 accounts for approximately 38.72% of the total of 86 *E. coli* isolates in addition to biofilm formation was detected in 69.7% of the sample that was positive for ST131 (Celebi et al., 2023).

*Klebsiella pneumoniae* ST258/ST512 is the predominant international vehicle for dissemination of the *blaKPC* carbapenemase gene, having driven the global spread of KPC-producing *K. pneumoniae* and related Enterobacteriaceae in multiple continents (Andrade et al., 2011; Baraniak et al., 2017). *K. pneumoniae* ST258 has been responsible for large-scale carbapenem-resistant outbreaks in the USA and Europe and is characterized by its capacity for large-scale chromosomal rearrangement and persistent MGE acquisition (Budia-Silva et al., 2024; Deleo et al., 2014).

*Staphylococcus aureus* USA300 is the most prevalent community-associated MRSA (CA-MRSA) lineage in the Americas, carrying SCCmec type IV and the Panton–Valentine leukocidin (PVL) genes. USA300 has demonstrated remarkable epidemiological success due to the combination of antibiotic resistance and enhanced virulence, resulting in severe skin and soft-tissue infections and, increasingly, healthcare-associated infections (Bonnstetter et al., 2007; Carrel et al., 2015).

A leading cause of carbapenem-resistant *Acinetobacter baumannii* International Clone 2 (IC2) infections in hospitalized patients. The majority of IC2 isolates carry bla_*OXA–23*_ disseminated via plasmids or transposons. IC2 demonstrates exceptional environmental persistence, facilitating intra- and inter-hospital transmission. Its tolerance of desiccation and disinfectants contributes substantially to its nosocomial success (Xie et al., 2025).

**FIGURE 2:**
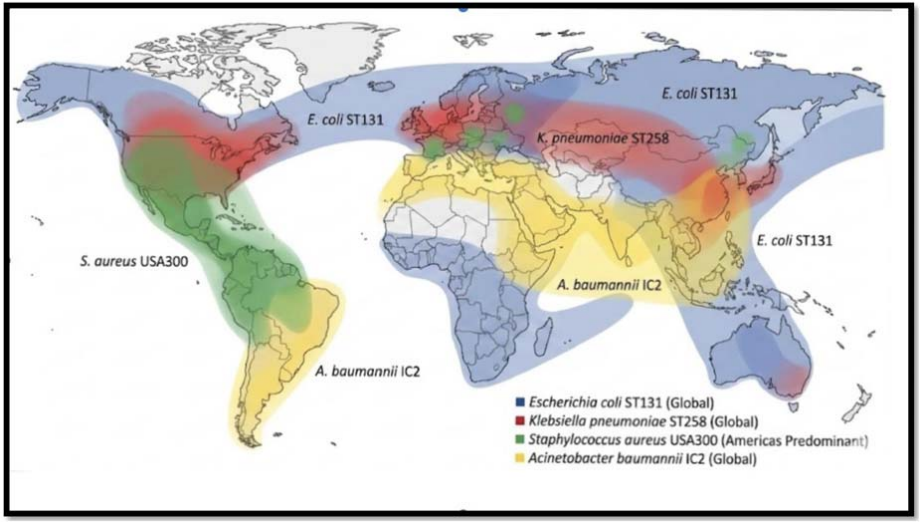
Global distribution of high-risk bacterial clones. A schematic world map illustrating the predominant geographical spread and overlap of key high-risk clones discussed (e.g., *E. coli ST131, K. pneumoniae ST258, S. aureus USA300, A. baumannii IC2*). Created in Gemini AI (2026) https://gemini.google.com.

International travel, interconnected healthcare systems, and variable infection control enforcement have accelerated the global dissemination of these high-risk clones. Molecular profiling — particularly WGS-based phylogenomic analysis—is indispensable for understanding their evolutionary trajectories and informing targeted containment strategies. Figure 2 illustrates the global distribution of high-risk bacterial clones worldwide.

## 3 From gene to function: decoding resistance phenotype

### 3.1 Expression regulation of resistance gene

**FIGURE 3:**
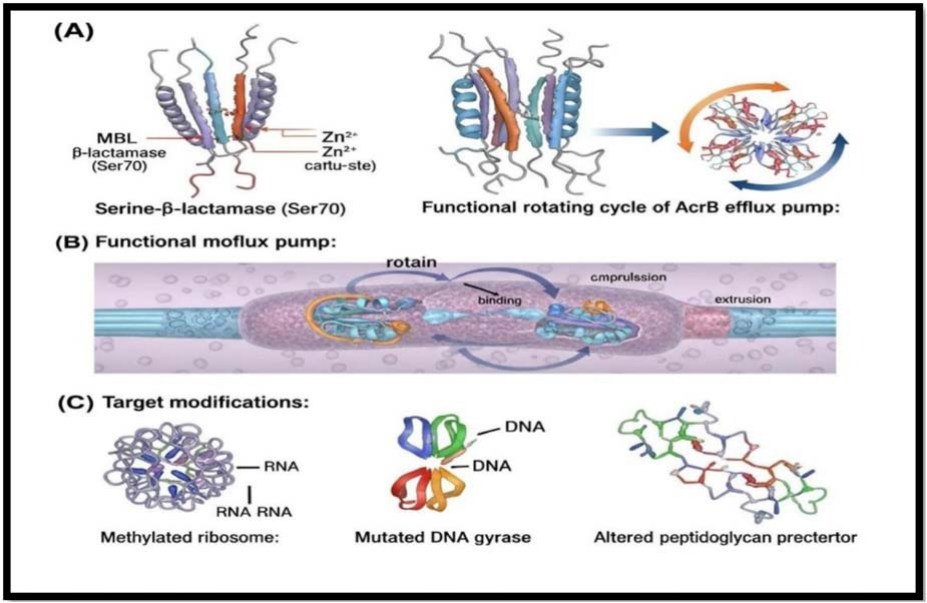
Schematic representation of key resistance mechanisms: **(A)** Catalytic sites of serine-β-lactamase (Ser70) and MBL (Zn^2+^), **(B)** functional rotating cycle of AcrB efflux pump, **(C)** target modifications: methylated ribosome, mutated DNA gyrase, and altered peptidoglycan precursor. Created in Gemini AI (2026) https://gemini.google.com.

Although the detection of a resistance gene is a prerequisite for phenotypic resistance, the relationship between genotype and observed susceptibility testing outcome is modulated by complex regulatory networks. Discordance between predicted genotype and measured minimum inhibitory concentration (MIC) is clinically significant and must be appreciated when interpreting molecular diagnostic results (Doyle et al., 2020; Wang et al., 2022). Figure 3 describes a schematic of the key resistance mechanisms.

*AmpC* β-lactamases are clinically important enzymes that, in species such as *Enterobacter cloacae*, *Citrobacter freundii*, and *Morganella morganii*, are encoded by chromosomal genes whose expression is inducible by β-lactam antibiotics via the conserved *AmpR*–*AmpD* regulatory circuit (Guérin et al., 2015; Hanson, 2003). In this context, β-lactam-induced cell-wall damage generates specific muropeptide signals that modify *AmpR* such that *ampC* transcription is transiently up-regulated; however, loss-of-function mutations in *ampD* result in constitutive, high-level *AmpC* expression, leading to treatment failure during third-generation cephalosporin therapy—a phenomenon termed “de-repression.” (Guérin et al., 2015; Hanson, 2003). By contrast, plasmid-encoded *AmpC* enzymes and many extended-spectrum β-lactamase (ESBL) genes (e.g., *bla*CMY-2, *bla*CTX-M) are typically expressed constitutively from strong promoters, conferring baseline resistance immediately upon acquisition and complicating the interpretation of susceptibility profiles in clinical isolates (Call et al., 2010; Li et al., 2015; Rizi et al., 2020).

### 3.2 Co-resistance and cross-resistance networks

Co-resistance occurs when multiple resistance genes are genetically linked on the same mobile genetic element, so selection for one antibiotic can co-select resistance to others. In *E. coli*, IncF plasmids have been described as carriers of *bla*_*CTX–M*_ and other resistance determinants, and aac(6′)-Ib-cr is common among ESBL-producing isolates, supporting the possibility of aminoglycoside-driven co-selection of ESBL-producing strains (Guillard et al., 2014; Li J. J. et al., 2015).

Cross-resistance occurs when a single mechanism confers resistance to multiple agents, either within a drug class (e.g., rpoB mutations conferring rifamycin class resistance) or across classes via efflux pump hyperexpression (Amsalu et al., 2020; Williams et al., 1998). Metagenomic analysis of environmental “resistomes” — the collection of resistance genes present within a given microbial community — has revealed that cross-resistance patterns can be predicted from gene co-occurrence networks, with implications for stewardship interventions targeting resistance-prone antibiotic classes (Li B. et al., 2015).

### 3.3 Fitness cost and compensatory evolution

Many resistance mechanisms carry a measurable fitness cost, including reduced growth rate, impaired virulence, or diminished competitive fitness. Without ongoing antibiotic selective pressure, these costs can lead to the gradual decline of resistant populations. However, compensatory mutations — arising at secondary genomic loci — frequently restore fitness while maintaining resistance, allowing resistant strains to persist and spread after antibiotic pressure is removed (Schulz zur Wiesch et al., 2010).

Mechanisms of compensation include second-site mutations restoring protein function, regulatory adjustments re-balancing metabolic homeostasis, and acquisition of new genetic material that mitigates the original fitness cost. The relevance of compensatory evolution is demonstrated in *Mycobacterium tuberculosis*, where compensatory mutations in ribosomal protein genes restore translational efficiency in rifampicin-resistant strains, and in *K. pneumoniae* ST258, where genomic plasticity enables rapid adaptation. Understanding compensatory evolution is critical for predicting the long-term stability of resistance in clinical and community settings and for assessing whether antibiotic cycling strategies can effectively reduce resistance burden (Eckartt et al., 2024; Ernst et al., 2020).

### 3.4 Structural and mechanistic basis of key resistance determinants

Translating genetic data into therapeutic insight requires structural and mechanistic understanding of resistance determinants. Below, key AMR mechanisms are described at the molecular level, with explicit linkage to the antibiotic classes and resistance phenotypes they confer.

#### 3.4.1 β-Lactamases: catalytic diversity and the ambler classification

β-Lactamases are classified into four Ambler molecular classes (A–D) based on their catalytic mechanism. Classes A, C, and D employ an active-site serine residue, while Class B enzymes (metallo-β-lactamases, MBLs) utilize zinc ions (Bush and Jacoby, 2010). Serine β-lactamases — including ESBLs such as CTX-M-15— form a covalent acyl-enzyme intermediate with the β-lactam ring via Ser70, followed by hydrolysis. Active-site mutations in CTX-M enzymes expand the substrate binding pocket, enabling hydrolysis of third-generation cephalosporins (Adamski et al., 2015; Ali et al., 2018). Carbapenemases (KPC, Class A; OXA-48, Class D) further adapt structurally to accommodate the carbapenem nucleus (Pitout et al., 2019). Metallo-β-lactamases (MBLs), on the other hand, utilize zinc-activated water molecules for β-lactam hydrolysis, conferring broad-spectrum resistance including to carbapenems, and are intrinsically resistant to all currently approved serine β-lactamase inhibitors (Page and Badarau, 2008).

#### 3.4.2 Efflux pumps: molecular architecture

Resistance-nodulation-division (RND) family efflux pumps (AcrAB-TolC in *E. coli*; MexAB-OprM in *P. aeruginosa*) are tripartite complexes spanning the bacterial envelope. The inner membrane transporter trimer undergoes proton motive force-driven conformational cycling, including access, binding, and extrusion, extruding structurally diverse substrates including fluoroquinolones, β-lactams, chloramphenicol, and tetracyclines. Hyperexpression due to regulatory mutations confers clinically relevant MDR (Venter et al., 2015; Yamasaki et al., 2023).

#### 3.4.3 Target modifications: atomic-scale interference

Three clinically important target modification mechanisms are described. (i) Ribosomal methylation: Erm methyltransferases methylate of adenine A2058 in 23S rRNA, sterically hindering binding of macrolide, lincosamide, and streptogramin B (MLS _B)_ antibiotics, conferring the MLS_B_ phenotype (Maravić, 2004). (ii) DNA gyrase/topoisomerase IV mutations: Point mutations within the quinolone resistance-determining region (QRDR) — most commonly Ser83Leu in GyrA — alter the geometry of the enzyme–DNA–drug complex, reducing fluoroquinolone binding affinity (Mehla and Ramana, 2016; Weigel et al., 2002). (iii) Vancomycin resistance: *VanA*-type systems encode a reprogrammed peptidoglycan biosynthesis pathway that substitutes D-Alanine-D-Lactate (D-Ala-D-Lac) for the normal D-Ala-D-Ala terminus of peptidoglycan precursors, eliminating the key hydrogen bond required for vancomycin binding and reducing affinity (Mainardi et al., 2008; Stogios and Savchenko, 2020).

## 4 Modern diagnostic platforms: translating insight into practice

The selection of an appropriate molecular diagnostic platform depends on the interplay between clinical urgency, required genomic resolution, available infrastructure, and cost. Diagnostic platforms can be conceptually organized along two axes: (i) turnaround time versus genomic breadth, and (ii) point-of-care suitability versus laboratory infrastructure requirement. Targeted PCR-based approaches occupy the rapid, low-complexity end of this spectrum, while WGS and metagenomics provide comprehensive genomic resolution at the cost of speed and infrastructure. Emerging platforms such as CRISPR-based diagnostics and nanopore sequencing are beginning to bridge these axes. Table 2 provides a structured comparison of all major platforms.

**TABLE 2:** Comparison of molecular diagnostic platforms for AMR detection.

Platform	Technology	Sensitivity/ specificity	Scalability	Clinical utility	Key advantage	Main limitation	Best use	Reference
Multiplex PCR	Endpoint PCR targeting multiple resistance genes	High for targeted genes	High — simple equipment; adaptable to most labs	Good for routine surveillance; guides empirical therapy	Low cost; simultaneous detection of multiple known AMR genes	Limited to predefined panel; no genomic or MGE context	Targeted surveillance of known resistance genes in clinical and environmental samples	Banerjee and Patel, 2023; Brazelton de Cardenas et al., 2021; Dung et al., 2024; Sigmund et al., 2020
qPCR	Probe-based real-time PCR	Very high for validated targets; must be assay-specific	High — widely available; closed-tube reduces contamination	Strong clinical utility for MRSA, carbapenemase detection	Rapid, quantitative results; closed-tube system reduces contamination risk	Limited multiplexing; targets are fixed and cannot detect novel mechanisms	Rapid clinical AMR detection (e.g., carbapenemases, MRSA)	Abramova et al., 2023; Silvester et al., 2025
Digital PCR	Partitioned endpoint PCR with absolute quantification	High sensitivity and precision for low-abundance targets	Low — high per-test cost limits large-scale deployment	Niche utility for heteroresistance monitoring	Absolute quantification without standard curve; excellent for minority variants	High per-test cost; not ideal for broad multi-target screening	Detection of heteroresistance and low-abundance resistance variants	Merino et al., 2022; Wu et al., 2022
WGS (Illumina)	Short read next generation sequencing (NGS)	High concordance with phenotypic AST for many pathogens; coverage dependent academic.	Moderate — requires infrastructure and bioinformatics capacity	High utility for outbreak investigation and national surveillance	Comprehensive resistome; plasmid/MGE context; phylogenomics and strain typing academic.	Slow TAT; bioinformatics and infrastructure demands; may miss truly novel mechanisms academic.	Reference laboratory use, outbreak investigation, and national/targeted AMR surveillance academic.	Bogaerts et al., 2021b; Bonvegna et al., 2022; Mitchell et al., 2022
Metagenomic sequencing	Shotgun NGS of all DNA in sample	Variable; depends on sequencing depth and target abundance academic.	Low — high cost and complex analysis limit routine use	Research utility; not yet standard in clinical practice	Culture independent; captures full resistome including uncultivable organisms	Resistance genes cannot always be confidently attributed to specific organisms; host DNA interference	Research on environmental and gut resistomes	Gweon et al., 2019; Serpa et al., 2022; Van Camp et al., 2023
Nanopore (MinION)	Long-read real-time sequencing	High accuracy and improving with chemistry and base calling updates academic.	Moderate — portable device but data analysis pipelines required	Promising for field outbreaks; resolves complex MGEs; emerging clinical use	Portable; real time data; resolves complex MGEs, repeats, and structural variants academic.	Higher raw error rate than Illumina short reads; data management and analysis pipeline complexity academic.	Field outbreak work; hybrid WGS for complex AMR elements (plasmids, transposons) academic.	Golparian et al., 2018; Greig et al., 2018
CRISPR-Dx (SHERLOCK/ DETECTR)	Cas12/Cas13 nucleic acid detection	Reported high sensitivity and attomolar LOD in prototype studies academic.	Potentially high — low-cost reagents; minimal equipment for POC	Emerging POC utility; not yet standardized for clinical use	Highly programmable; potential for low cost, decentralized, point of care (POC) use academic.	Clinical validation and multiplexing at scale still ongoing; not yet standardized academic	Resource limited and low-income settings; rapid POC AMR detection under development academic	Lau et al., 2025; Tang et al., 2026; Zhang X. et al., 2024

AMR, antimicrobial resistance; POC, point of care; TAT, turn around time; AST, antimicrobial susceptibility testing; WGS, whole genome sequencing; MRSA, methicillin resistant *Staphylococcus aureus*.

### 4.1 Targeted gene detection methods

Multiplex PCR enables simultaneous detection of multiple resistance gene targets in a single reaction. Commercial platforms such as the Check-MDR CT103 assay detect clinically important ESBL, carbapenemase, and *AmpC* gene families directly from bacterial isolates (Chavda et al., 2016; Cunningham et al., 2016). Sensitivity for included targets generally exceeds 95% where clinical validation data are available. The principal limitation is panel fixity where only resistance genes known at the time of assay design can be detected, and no information on genomic context — such as plasmid replicon type or co-resistance gene carriage — is generated (Banerjee and Patel, 2023).

Quantitative PCR (qPCR) or real-time PCR offers rapid, quantitative results with high specificity due to probe-based detection in a closed-tube format, minimizing the risk of amplicon contamination. The Cepheid Xpert Carba-R assay detects the five major carbapenemase gene families (blaKPC, blaNDM, blaVIM, blaIMP-1, and blaOXA-48). Sensitivity and specificity for these five major carbapenemase families exceed high amount in published validation studies (Ko et al., 2019). The limitation is narrow multiplexing capacity; qPCR is not suited to comprehensive resistance profiling requiring simultaneous detection of targets.

Digital PCR (dPCR) partitions a single reaction into thousands of independent micro-reactions, enabling absolute quantification of target gene copies without an external standard curve. This confers exceptional sensitivity for low-abundance resistance genes in mixed or hetero-resistant populations (Sedlak and Jerome, 2013). dPCR is particularly suited to detecting minority resistance variants that would be missed by bulk qPCR, a situation relevant to hetero-resistance in *S. aureus* or ESBL-producing minority clones in bacteraemia. Current limitations include high cost per test and unsuitability for high-throughput multi-target screening (Zhang H. et al., 2024).

### 4.2 Sequencing-based approaches

Whole-Genome Sequencing (WGS) provides comprehensive identification of both characterized and novel resistance genes, plasmid replicon types, MGE context, and phylogenetic relationships — none of which are available from targeted PCR methods. Analysis against curated databases (CARD, ResFinder) enables prediction of resistance phenotypes (Bortolaia et al., 2020; Shelburne et al., 2017). Published studies report high concordance between WGS-predicted genotype and AST phenotype for most well-characterized resistance mechanisms (e.g., *mecA*/MRSA, *vanA*/VRE, *blaKPC*), whereas agreement is lower for complex regulatory mechanisms such as efflux hyperexpression or porin loss (He et al., 2026; Sharma et al., 2024).

Metagenomic Sequencing enables culture-independent characterization of the complete microbial community and its resistome directly from clinical specimens. This approach can identify resistance genes from non-culturable organisms and characterize the broader genomic context of resistance (Willmann and Peter, 2017). A critical limitation is the inability to definitively attribute a detected resistance gene to a specific bacterial host within a mixed sample, potentially confounding interpretation. The high proportion of host DNA in clinical specimens further reduces effective sensitivity for bacterial targets. Bioinformatic complexity and cost remain significant barriers to routine clinical implementation (Ji et al., 2020).

Nanopore Sequencing (e.g., MinION) generates long reads in real time from a portable device, enabling rapid near-patient genomic investigations. The ability to resolve complex MGE structures — including repetitive elements within plasmids that are difficult to assemble from short Illumina reads — is a key advantage for AMR genomics (Peng et al., 2025; Zhao et al., 2023). Published studies include rapid outbreak investigation and real-time resistome characterization in resource-limited settings during field deployments, for example, nanopore-based protocols that map resistance-gene-carrying plasmids and outbreak-linked *Staphylococcus aureus* and *Klebsiella pneumoniae* isolates (Avershina et al., 2022; Ferreira et al., 2021; Peter et al., 2020). Despite improvements in raw read accuracy with recent chemistry updates, error rates in homopolymeric regions remained higher than Illumina, leading to occasional frameshifts in AMR gene sequences (Gargis et al., 2019).

### 4.3 Rapid integrated panels for clinical use

Sample-to-answer automated systems integrate extraction, amplification, and detection into sealed single-use cartridges, minimizing hands-on time, operator variability, and contamination risk (Wang et al., 2021). These platforms are optimized for clinical settings requiring rapid, actionable results without specialist laboratory infrastructure.

BioFire FilmArray panels detect pathogens and key resistance markers (*mecA*, *vanA/B*, *blaKPC*) from positive blood cultures in under 1 h, enabling targeted antibiotic de-escalation up to 24 h earlier than culture-based methods (Fhooblall et al., 2016). Meta-analytic evidence demonstrates that this time reduction translates into improved antibiotic selection and reduced broad-spectrum antibiotic exposure when integrated with pharmacist-led stewardship Deresinski (2016).

Multiplex PCR-based panels for pneumonia (BioFire FilmArray Pneumonia Panel, Curetis Unyvero) and bloodstream infections (GenMark ePlex) provide semi-quantitative resistance marker detection from respiratory specimens and blood cultures (Buchan et al., 2020; Caspar et al., 2024; Mizusawa and Carroll, 2023). However, all rapid panel systems share important limitations: panel fixity prevents detection of emerging or unexpected resistance mechanisms; closed reagent systems create supply chain dependency; and the high per-test cost may not be cost-effective in low-prevalence settings (Tansarli et al., 2025).

### 4.4 Emerging point-of-care technologies

Clustered regularly interspaced short palindromic repeats-based Diagnostics are platforms based on the collateral cleavage activity of Cas12 (DETECTR) and Cas13 (SHERLOCK) enzymes enable highly sensitive nucleic acid detection using fluorescent or lateral flow readouts (Mustafa and Makhawi, 2021). Published analytical performance data for CRISPR-based diagnostics demonstrate sensitivities exceeding 95% for targeted resistance genes, with detection limits reaching the attomolar range in optimized *in vitro* assays (Wang et al., 2025).

In proof-of-concept studies, CRISPR-Cas12a-based detection of *blaKPC* and *blaNDM* from spiked clinical specimens has been demonstrated at low cost, with lateral flow readout amenable to deployment in LMICs (Hyeon et al., 2026). The major remaining challenges are multiplexing capacity, the need for an upstream nucleic acid extraction step, and the requirement for large-scale prospective clinical validation studies before regulatory approval. Programmatic production of guide RNAs is straightforward, enabling rapid assay adaptation to emerging resistance variants (Zhou et al., 2026).

Microfluidics and Lab-on-a-Chip are miniaturized systems that integrate cell lysis, nucleic acid extraction, amplification, and detection into a single disposable cartridge, enabling near-patient testing with minimal reagent volumes. A key engineering challenge is achieving sufficiently sensitive detection in a miniaturized format, particularly for low-bacterial-burden specimens such as cerebrospinal fluid. Commercial platforms represent early-generation microfluidic AMR diagnostics (Cai et al., 2026; Liu et al., 2022; Obino et al., 2021).

Surface-Enhanced Raman Spectroscopy (SERS) generates unique molecular vibration fingerprints from bacterial cells, enabling label-free identification. When coupled to machine-learning classifiers, SERS has demonstrated species identification and early-stage resistance profiling for β-lactamase-producing organisms (Ciloglu et al., 2021; Hassanain et al., 2022). The critical challenge for clinical translation remains the development of reproducible, batch-consistent SERS substrates and the establishment of large, externally validated spectral reference databases. Current performance benchmarking is predominantly from single-center studies using standardized reference strains rather than diverse clinical isolates (Lee et al., 2026; Pilot et al., 2019).

## 5 Integrating molecular data into clinical decision-making: bridging the translational gap

The generation of molecular data is just the beginning. The real value of molecular data is in its conversion to intervention for public health and clinical purposes. There are significant operational and interpretive challenges associated with the integration of this data.

### 5.1 Impact on antibiotic stewardship

Although rapid molecular diagnostics may allow for quicker targeted therapies and de-escalation, implementation within appropriate stewardship programs is key for assessing real-world benefits.

The clinical value of molecular diagnostics is realized only when results are effectively integrated into stewardship frameworks. Evidence from multiple randomized and observational studies demonstrates that rapid molecular testing alone is insufficient — the magnitude of clinical benefit is directly dependent on the presence of co-interventional stewardship support at the time of result delivery (De Waele and Boelens, 2024; Timbrook et al., 2017).

A meta-analysis study to determine the clinical impact of rapid diagnostic tests (RDTs) in bloodstream infection testing embedded within antimicrobial stewardship programs (ASPs) by using rapid panels (BioFire, Accelerate) has shown to have a benefit in identifying a more timely initiation of an effective antibiotic therapy while simultaneously decreasing broad-spectrum antibiotic use, proving the use of RDT with ASP may lead to a survival benefit even when introduced in settings already adopting effective ASP in association with conventional blood culture (BC; Peri et al., 2024). Mortality benefit has been demonstrated in some but not all studies, with the greatest benefit observed in high-risk populations (e.g., immunocompromised patients, patients with carbapenem-resistant organisms).

In respiratory infections, the BioFire FilmArray Pneumonia Panel provides semi-quantitative pathogen and resistance marker data from sputum or bronchoalveolar lavage (BAL) within approximately 1 h, including detection of *mecA*, *blaKPC*, and *blaNDM*. For healthcare-associated pneumonia (HAP) and ventilator-associated pneumonia (VAP), molecular result-guided de-escalation has been shown to reduce carbapenem and antifungal use without adverse patient outcomes. For community-acquired pneumonia (CAP), however, the clinical benefit of molecular diagnostics remains debated, as detection of a pathogen does not reliably alter empirical therapy in the majority of cases (Chen et al., 2017; Falsey et al., 2024; Murphy et al., 2020; Poole et al., 2022).

Rapid PCR-based screening of rectal swabs for Carbapenemase-producing Enterobacterales (CPE) (e.g., Xpert Carba-R) enables pre-emptive contact isolation of colonized patients on hospital admission, preventing nosocomial CPE transmission (Jin et al., 2020, 2021). Modeling studies suggest this approach is cost-effective in high-prevalence settings (Toumi et al., 2025). Successful implementation requires clear institutional protocols for result escalation, dedicated infection control resources, and clinician education to prevent “alert fatigue” (Fallon et al., 2024).

### 5.2 Real-time outbreak investigation and genomic surveillance

Whole-Genome Sequencing (WGS) enables high-resolution strain typing, allowing differentiation of true nosocomial transmission clusters from coincidental co-detection of phenotypically similar but genetically unrelated isolates (Uelze et al., 2020). On the other hand, the core genome of single nucleotide polymorphism (SNP) threshold of ≤5–10 SNPs is widely used to define epidemiologically linked clusters, though laboratory- and species-specific thresholds are required (Stimson et al., 2019).

Genomic epidemiology tools can link geographically dispersed human cases to common food or environmental sources, as demonstrated in international investigations of *Listeria* and *Salmonella* outbreaks (Deng et al., 2016). Initiatives of national and global network including EURGen-Net, and Pathogenwatch provide standardized, automated analysis pipelines and real-time data-sharing infrastructures, lowering barriers to genomic surveillance globally (Struelens et al., 2024).

### 5.3 Implementation in low- and middle-income countries

The translational benefits of molecular diagnostics are most urgently needed in LMICs, which bear the greatest burden of AMR mortality, yet face the greatest barriers to implementation (Ikhimiukor et al., 2022). Barriers include high capital costs for sequencing platforms and molecular diagnostic equipment; lack of uninterrupted electricity supply and cold-chain logistics; insufficient numbers of trained clinical microbiologists and bioinformaticians; and the absence of reimbursement frameworks for molecular tests in many national healthcare systems (Zhang et al., 2026).

Tiered diagnostic approaches are therefore recommended for LMIC settings: a first-tier consisting of validated CRISPR-based or loop-mediated isothermal amplification (LAMP) assays targeting the highest-priority resistance mechanisms (carbapenemases, ESBLs, MRSA) with minimal infrastructure requirements; a second-tier involving qPCR-based platforms for reference hospitals; and WGS-based surveillance reserved for national reference centers and outbreak investigations (NIHR Global Health Research Unit on Genomic Surveillance of AMR, 2020; Ortiz-Cartagena et al., 2025; Zhang et al., 2026). This model mirrors the tiered approach adopted for tuberculosis diagnostics under the WHO End TB Strategy (Uplekar et al., 2015).

Innovative financing mechanisms—analogous to the AMR Action Fund for antibiotic development, and the FIND and UNITAID models for diagnostics procurement—are urgently needed to improve equitable access (Singer et al., 2020). The WHO Global Antimicrobial Resistance and Use Global antimicrobial resistance and use surveillance System (GLASS) and regional networks such as the WHO Extended-Spectrum Beta-Lactamase (ESBL) network provide a framework for graduated capacity building in LMIC settings (Ajulo and Awosile, 2024).

## 6 Challenges and limitations

The promise of molecular AMR diagnostics must be assessed against a series of practical, economic, and regulatory challenges that currently limit their impact in both high-income and low-income settings.

### 6.1 Technical and interpretative hurdles

Genotype–Phenotype Discordance: A critical challenge for molecular AMR diagnostics is the imperfect correlation between resistance gene presence and measured minimum inhibitory concentration (MIC). Sources of discordance include: (i) silent mutations, truncated open reading frames, or gene fragments that prevent functional resistance protein production; (ii) weak or absent promoters reducing expression below the clinically relevant threshold (e.g., certain *bla*_*NDM*_ alleles with borderline MICs); (iii) porin loss or outer membrane permeability changes conferring carbapenem non-susceptibility in the absence of a detectable carbapenemase gene; and (iv) resistance mediated by regulatory mutations with no corresponding sequence-based marker in current databases. Conversely, gene detection does not guarantee clinical resistance: some ESBL-gene-carrying isolates may have MICs below European Committee on Antimicrobial Susceptibility Testing and Clinical and Laboratory Standards Institute (EUCAST/CLSI) susceptible breakpoints due to poor promoter activity (Kowalska-Krochmal and Dudek-Wicher, 2021; Turnidge and Paterson, 2007; Yee et al., 2021). These discordances require that molecular results be interpreted in conjunction with phenotypic AST for clinical decision-making.

Sequence similarity-based tools such as CARD (Comprehensive Antibiotic Resistance Database) and ResFinder cannot identify resistance genes lacking homology to characterized reference sequences. Functional metagenomics—involving cloning of environmental or clinical DNA into susceptible host strains followed by resistance screening—can identify genuinely novel determinants but is labor-intensive and not amenable to routine clinical use (Alcock et al., 2020; Gschwind et al., 2023). Due to a lack of standardization of bioinformatic pipelines, thresholds of quality control, and formats for reporting, the comparability of results of WGS and metagenomic studies from different laboratories remains limited (Bogaerts et al., 2021a; Lavrichenko et al., 2025).

### 6.2 Economic, infrastructure, and equity barriers

For AMR detection, the equipment, consumables, service contracts, and software licensing associated with WGS, and rapid molecular panel platforms impose substantial financial burdens. Although WGS has been successfully applied to identify drivers of AMR burden for most laboratories in sub-Saharan Africa and South Asia; however, due to its high cost and complexity, WGS is currently mainly carried out more in high-income countries (NIHR Global Health Research Unit on Genomic Surveillance of AMR, 2020).

Advanced molecular diagnostics require expertise in molecular biology, sequencing technologies, and bioinformatics—competencies that are in critically short supply in clinical microbiology laboratories worldwide. The WHO estimates a substantial global shortage of health workers, with microbiology laboratory capacity disproportionately underdeveloped in low- and middle-income countries (LMICs; Aluttis et al., 2014; Kozlakidis et al., 2020). The COVID-19 pandemic exposed severe vulnerabilities in the global supply chains for molecular diagnostic reagents, leading to widespread test shortages (Cornish et al., 2023; Stanton et al., 2022). As a result, it might lead to disrupting both AMR surveillance and clinical diagnostic capacity. Building regional reagent manufacturing capacity and diversifying supplier networks are essential resilience measures.

### 6.3 Quality assurance and regulatory pathways

External quality assurance (EQA) is a process in which laboratories receive standardized samples from an external provider to assess and compare the accuracy and consistency of their test results against a reference or peer group (Kostyanev et al., 2025). While EQA is critical for monitoring AMR diagnostic quality, its effectiveness is limited by narrow panel coverage, inconsistent follow-up of EQA failures, technical heterogeneity in AST and molecular methods, and unequal access across regions (Abegaz et al., 2025).

Regulatory fragmentation represents a substantial barrier to the global rollout of validated molecular AMR tests. Divergent regulatory requirements across the USA (FDA), European Union (CE-IVD), and national regulatory bodies in LMICs result in significant delays—often several years—between initial development and approval in high-burden settings (Gupte et al., 2025; Lucien and Cantave, 2026). Collaboration of international diagnostic regulatory frameworks, as advocated by the International Medical Device Regulators Forum (IMDRF), is urgently required.

## 7 Future directions

### 7.1 Emerging technologies and approaches

Integration AI and Machine Learning systems are being applied to predict AMR phenotypes directly from raw genomic data, bypassing the need for manually curated resistance gene databases (Arnold et al., 2025; Kim et al., 2022). Tools such as DeepARG is a prominent deep learning-based tool designed to improve the identification and annotation of antibiotic resistance genes (ARGs) in metagenomic data (Arango-Argoty et al., 2018). Importantly, Machine learning (ML)-based phenotype prediction offers a significant advantage over traditional gene-catalogue approaches by identifying resistance mechanisms arising from complex polygenic and non-linear genetic interactions (Bonet et al., 2024). Validated ML models are increasingly capable of matching or approaching the accuracy of conventional phenotypic AST for *M. tuberculosis* and *E. coli*, often providing results significantly faster (Sturm et al., 2024; Vocat et al., 2023).

On the other hand, microfluidic isolation of bacterial cells is a transformative approach, enabling the characterization of phenotypic heterogeneity and antibiotic resistance that is often hidden by traditional bulk methods (Agnihotri et al., 2025). Furthermore, engineered bacteriophages incorporating reporter constructs and synthetic gene circuits represent a promising next-generation diagnostic platform for addressing antimicrobial resistance (AMR), offering high specificity and rapid phenotypic susceptibility testing (Meile et al., 2020; Zelcbuch et al., 2021).

### 7.2 Toward personalized antibiotic therapy

Future personalized antibiotic therapy will integrate detailed pathogen resistance profiles, including mobile resistance elements and regulatory mutations with patient pharmacogenomic data governing antibiotic pharmacokinetics and pharmacodynamics, and microbiome composition data informing collateral damage to commensal flora. This multi-data-type approach will enable optimized dosing regimens and combination therapy strategies tailored to the individual patient and pathogen (Cusumano et al., 2025; Shaman, 2024; Tran and Dahlin, 2024).

The ongoing development of β-lactamase inhibitors, efflux pump inhibitors, and permeability enhancers represents a complementary strategy to restore the efficacy of established antibiotics against resistant strains (Belay et al., 2024; Li et al., 2026). Mathematical modeling integrating real-time genomic surveillance data can further inform antibiotic cycling and mixing strategies at hospital and regional levels (Knight et al., 2019).

### 7.3 Policy implications and the imperative for global cooperation

Professional societies must develop evidence-based guidelines integrating molecular test results into clinical pathways, defining optimal use cases and performance thresholds for cost-effective deployment (Ringborg et al., 2026). Innovative financing models — analogous to the AMR Action Fund for antibiotic development — are needed to bridge the diagnostic equity gap and improve access to molecular AMR testing in LMICs (Anderson et al., 2023). The WHO Global Action Plan on AMR provides a strategic framework; however, binding international agreements on antimicrobial use in agriculture, standardized surveillance data sharing, and transparent genomic data governance are required for a coordinated global response (World Health Organization [WHO], 2026).

## 8 Conclusion

The molecular revolution has fundamentally transformed our understanding of antimicrobial resistance and our capacity to diagnose it. The progressive characterization of resistance gene repertoires, the mechanistic decoding of their functional consequences, and the translation of these insights into a new generation of molecular diagnostic platforms represent some of the most consequential advances in contemporary infectious disease medicine. This review has traced this trajectory — from the molecular biology of the mobilome to the practical implementation challenges facing molecular diagnostics in clinical and public health settings.

Technological advances alone, however, are insufficient to address the AMR crisis. The greatest translational challenges now lie in bridging complex genomic data to simple clinical decisions, ensuring equitable global access to validated diagnostics, building sustainable laboratory capacity, and enforcing the collaborative governance required by a truly integrated One Health approach.

The continued development of AI-enabled genomics, point-of-care platforms, and personalized medicine will progressively improve the precision with which AMR is managed. Realizing this potential will require not only sustained investment in technology development, but also commensurate investment in human capital, laboratory infrastructure, and international cooperative frameworks. The molecular approach to AMR represents not merely a technological transition, but a fundamental evolution in the global strategy to preserve the efficacy of antimicrobials for future generations. Its success will ultimately be measured not in publications or patents, but in lives saved and antibiotic-days averted — outcomes that demand urgency, equity, and global solidarity.
